# A systematic review and meta-analysis of digital application use in clinical research in pain medicine

**DOI:** 10.3389/fdgth.2022.850601

**Published:** 2022-11-02

**Authors:** Ashish Shetty, Gayathri Delanerolle, Yutian Zeng, Jian Qing Shi, Rawan Ebrahim, Joanna Pang, Dharani Hapangama, Martin Sillem, Suchith Shetty, Balakrishnan Shetty, Martin Hirsch, Vanessa Raymont, Kingshuk Majumder, Sam Chong, William Goodison, Rebecca O’Hara, Louise Hull, Nicola Pluchino, Naresh Shetty, Sohier Elneil, Tacson Fernandez, Robert M. Brownstone, Peter Phiri

**Affiliations:** ^1^ University College London Hospitals NHS Foundation Trust, London, United Kingdom; ^2^ Nuffield Department of Primary Care Health Sciences, University of Oxford, Oxford, United Kingdom; ^3^ Department of Statistics and Data Science, Southern University of Science and Technology, Shenzhen, China; ^4^ Alan Turing Institute, London, United Kingdom; ^5^ Queen Square Institute of Neurology, University College London, London, United Kingdom; ^6^ Research & Innovation Department, Southern Health NHS Foundation Trust, Southampton, United Kingdom; ^7^ Department of Women and Children’s Health, Liverpool Women’s NHS Foundation, Liverpool, United Kingdom; ^8^ Praxisklinik am Rosengarten Mannheim, Saarland University Medical Centre, Homburg, Germany; ^9^ Eötvös Loránd University, Budapest, Hungary; ^10^ Academy of High Education, Sri Siddhartha University, Tumkur, India; ^11^ Oxford University Hospitals NHS Foundation Trust, Gynaecology, Oxford, United Kingdom; ^12^ Department of Psychiatry, University of Oxford, Oxford, United Kingdom; ^13^ University of Manchester NHS Foundation Trust, Gynaecology, Manchester, United Kingdom; ^14^ Robinson Research Institute, University of Adelaide, Adelaide, Australia; ^15^ University of Geneva, Gynaecology, Geneva, Switzerland; ^16^ Department of Orthopedics, M.S. Ramaiah Medical College, Bangalore, India; ^17^ Chronic Pain Medicine, Royal National Orthopaedic Hospital, London, United Kingdom; ^18^ Primary Care, Population Sciences and Medical Education Division, University of Southampton, Southampton, United Kingdom

**Keywords:** chronic pain, pain management, digital app, digital medicine, mHealth

## Abstract

**Importance:**

Pain is a silent global epidemic impacting approximately a third of the population. Pharmacological and surgical interventions are primary modes of treatment. Cognitive/behavioural management approaches and interventional pain management strategies are approaches that have been used to assist with the management of chronic pain. Accurate data collection and reporting treatment outcomes are vital to addressing the challenges faced. In light of this, we conducted a systematic evaluation of the current digital application landscape within chronic pain medicine.

**Objective:**

The primary objective was to consider the prevalence of digital application usage for chronic pain management. These digital applications included mobile apps, web apps, and chatbots.

**Data sources:**

We conducted searches on PubMed and ScienceDirect for studies that were published between 1st January 1990 and 1st January 2021.

**Study selection:**

Our review included studies that involved the use of digital applications for chronic pain conditions. There were no restrictions on the country in which the study was conducted. Only studies that were peer-reviewed and published in English were included. Four reviewers had assessed the eligibility of each study against the inclusion/exclusion criteria. Out of the 84 studies that were initially identified, 38 were included in the systematic review.

**Data extraction and synthesis:**

The AMSTAR guidelines were used to assess data quality. This assessment was carried out by 3 reviewers. The data were pooled using a random-effects model.

**Main outcome(s) and measure(s):**

Before data collection began, the primary outcome was to report on the standard mean difference of digital application usage for chronic pain conditions. We also recorded the type of digital application studied (e.g., mobile application, web application) and, where the data was available, the standard mean difference of pain intensity, pain inferences, depression, anxiety, and fatigue.

**Results:**

38 studies were included in the systematic review and 22 studies were included in the meta-analysis. The digital interventions were categorised to web and mobile applications and chatbots, with pooled standard mean difference of 0.22 (95% CI: −0.16, 0.60), 0.30 (95% CI: 0.00, 0.60) and −0.02 (95% CI: −0.47, 0.42) respectively. Pooled standard mean differences for symptomatologies of pain intensity, depression, and anxiety symptoms were 0.25 (95% CI: 0.03, 0.46), 0.30 (95% CI: 0.17, 0.43) and 0.37 (95% CI: 0.05, 0.69), respectively. A sub-group analysis was conducted on pain intensity due to the heterogeneity of the results (*I*^2^ = 82.86%; *p* = 0.02). After stratifying by country, we found that digital applications were more likely to be effective in some countries (e.g., United States, China) than others (e.g., Ireland, Norway).

**Conclusions and relevance:**

The use of digital applications in improving pain-related symptoms shows promise, but further clinical studies would be needed to develop more robust applications.

**Systematic Review Registration:**

https://www.crd.york.ac.uk/prospero/, identifier: CRD42021228343.

## Introduction

High-quality research data generated by scientifically robust study designs, improved use of clinical data, and the development of cost-effective healthcare models can change how medicine is practiced in the modern world. Digital medicine (DM), wherein multimodal and multidimensional digital tools are used to intervene in accessing and providing healthcare, is now a fundamental part of these drivers of change. Digital medicine describes a field, concerned with the use of technologies as tools for measurement, and intervention in the service of human health (1). Digital medicine products are driven by high-quality hardware and software that support the practice of medicine broadly, including treatment, recovery, disease prevention, and health promotion for individuals and across populations. Digital medicine products can be used independently or in concert with pharmaceuticals, biologics, devices, or other products to optimize patient care and health outcomes. Digital medicine empowers patients and healthcare providers with intelligent and accessible tools to address a wide range of conditions through high-quality, safe, and effective measurements and data-driven interventions. As a discipline, digital medicine encapsulates both broad professional expertise and responsibilities concerning the use of these digital tools. Digital medicine focuses on evidence-generation to support the use of these technologies.

Despite relative growth profoundly impacting gross economic improvement, “*bench to bedside*” pathways still take considerable time (2, 3). Equally robust research evaluations have not kept pace with a growing global population, although, the intellectual and healthcare evolution has modernised clinical practice by way of clinical research. Existing clinical evidence and incorporation of information technology has led to more prominent use of DM. A fundamental aspect of DM is to improve and promote evidence-based medicine (EBM) and/or evidence-based practices (EBP) within clinical and healthcare frameworks, underpinned by data science and technologies.

The future of digital medicine involves evolution of Artificial Intelligence (AI) based systems that may allow capture and dissemination of information in possible formats as below:

• Data Flows: Data can come in by the minute or millisecond (e.g., continuous glucose monitoring, heart rate information)
• Algorithmic Data: Results produced from algorithms run on large samples of data (e.g., genomic sequencing).
• Algorithmic Machine-Shared data: An algorithm shares a digital result. Limited context exists for a human to correct false positives/negatives in real-time.

The field of pain medicine in adults is a particularly challenging area of clinical practice for many reasons, including subjectivity associated with patient-reported outcomes and management of symptomatology with limited information on pathophysiology (4, 5). Considering this uncertainty, attempts by clinicians to categorise pain and decide on treatment interventions (Supplementary Table S1), could benefit from the concepts of DM and its associates of EBM and EBP. Pain is often the commonest symptom that patients present with in outpatient clinics. The need for individualised care based on generalisable research is complicated by wide variables, subjective nature, and inherent bias which provide a unique set of challenges for a simple protocol to work. The use of cognitive technology such as those that are AI-based, in delivering personalised care, based on available evidence, is therefore an attractive proposition for pain medicine.

The ability to modify behaviour may have implications for chronic disease management. For example, according to the United States Center for Disease Control and Prevention (CDC), there are currently 96 million prediabetic patients in the US. As would be of importance, preventing those individuals from advancing to full-blown diabetes through drug and/or device therapies or behavioral modifications would have a huge impact on morbidity and health economics. Apps that allow early intervention and monitoring of prediabetes could start to shift medical practice from treatment to prevention and early intervention. Novartis (Basel) aimed at developing a contact lens that can monitor a person's blood sugar levels (6), which could be applicable for both diabetics and, more generally, for alerting a user to the presence of a prediabetic state.

Pain medicine has been identified as a specialty that would vastly benefit from the personalisation of care (7). A current example of this need is the variable efficacy of pharmacotherapy in relieving chronic pain. Opioids, for instance, have been routinely used to treat chronic pain syndromes, despite only modest evidence for their use (8). This has the potential for significant harm in patients where it has been used inappropriately and may have influenced factors that led to the Opioid Crisis globally (65, 66), especially so in the United States and UK. Traditional pain evaluation methods are vulnerable to recall error and bias as they rely on retrospective reporting of pain variations (9). Pain perception combined with measuring functional changes and physiological parameters affected by pain are important secondary outcome data to assess efficacy. Methods demanding frequent, repeated pain evaluation and pain-associated features are required to formulate chronic pain management strategies (10, 11). This approach was previously hindered both by the resources required for such vast data collection, and the complexity of the statistical analysis required to interpret the resulting datasets (12).

Machine learning (ML) automatically processes large datasets and uses this to formulate informed predictions without the need for human intervention (13). ML algorithms continually update themselves with new information to ensure the most accurate and up-to-date trends are forecasted (14). It can be difficult for ML models to process complex datasets but techniques, such a data pre-processing, allow prediction models to transform datasets into predictions (15). Such models are widely used within environmental research, to assess and predict trends of climate change and air pollution however, ML has the scope to be applied to healthcare as well (15, 16). Within cardiology, a Rank-Based Deep Convolutional Neural Network is being successfully used to assess and classify electrocardiograms with a 96.7% success rate (17). ML is very commonly used in antenatal care throughout pregnancy and predict childbirth procedures, as well as highlight any complications (18). With its successful application to various fields within healthcare, it could prove useful for ML technologies to implemented into pain management.

To advance DM concepts and their use in pain medicine research, it is imperative to assess the global regulatory sphere. Over the last decade, a plethora of legislations and regulatory guidelines around DM have been developed by the World Health Organisation (WHO) (19), Medicines and Healthcare products Regulatory Agency (MHRA) (20), Food and Drug Administration (FDA) (21) and National Institute for Health and Care Excellence (NICE) (22) (Supplementary Table S2). However, there are complexities around evaluating AI-based applications that fall under the category of DM. This includes those using algorithms based on ML models that may be categorised as a medical device. Furthermore, development of AI applications requires documentary evidence that the planning, designing, and development phases meet the globally accepted International Organisation for Standardisation (ISO) standards. In order to achieve ISO standards, a high proficiency of conformances should be maintained by the research group responsible for developing the intervention that could be mass produced. As part of this standardisation process, the intervention may undergo several non-conformity assessments as well as vigorous testing and validation prior to being deployed.

**Figure 1:**
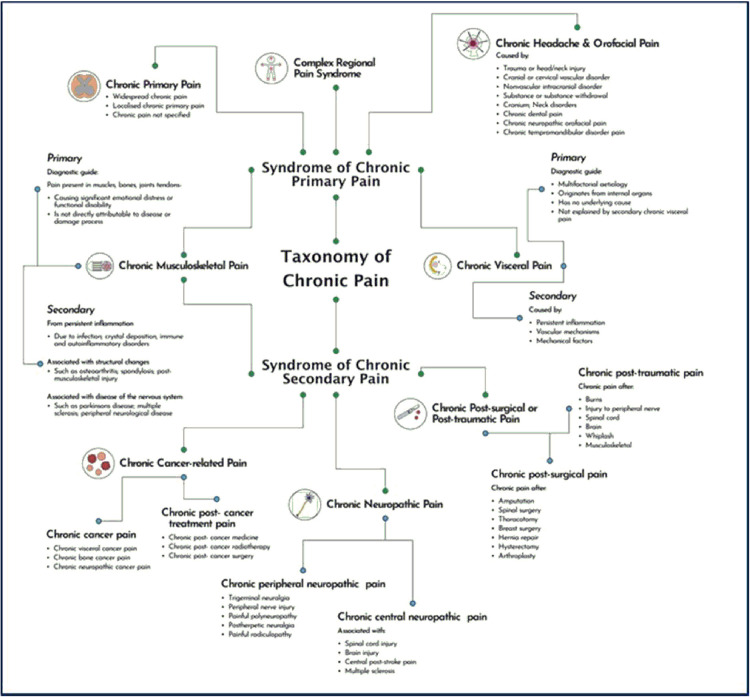
Chronic pain classification tree (CPCT).

**Figure 2:**
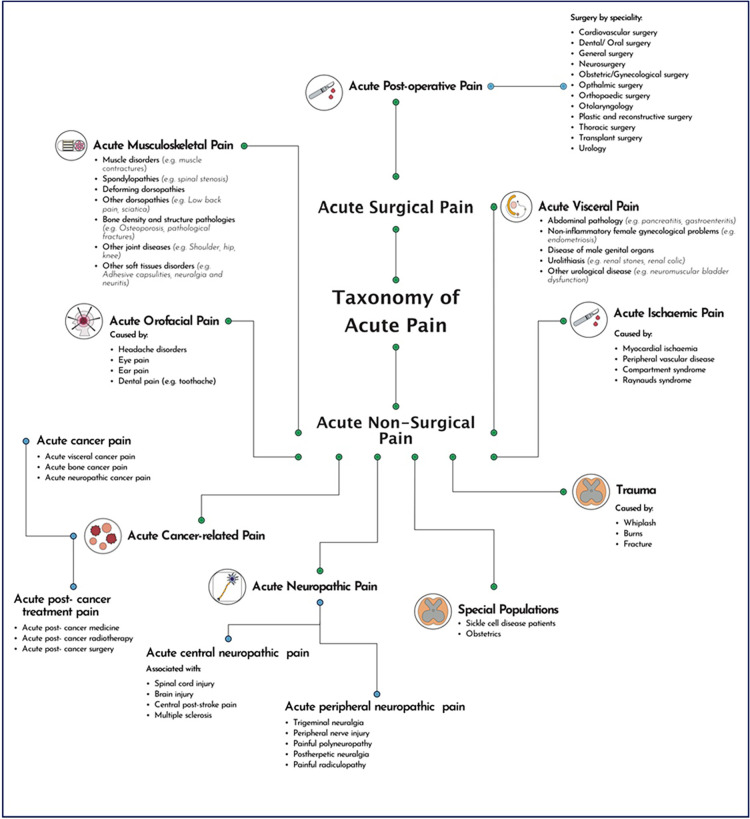
Acute pain classification tree.

The regulatory and standards required for novel innovations are also dependent on the disease classification. The current classification of chronic and acute pain conditions (Figures 1, 2 respectively) employs the guidelines published by the International Association for the Study of Pain. Clinicians evaluating both chronic and acute chronic pain are considering changes to guidelines to provide better diagnoses and improve outcomes for patients. Advancements in the understanding of the pathophysiology of acute and chronic pain have resulted in effective pharmacological approaches to sub-populations of patients.

A critical step of DM is the development of digital tools using large sets of datasets and aggregated data to create novel paradigms of care. This is also referred to as evidence-based digital medicine which uses EBM concepts. To disperse these paradigms, computer programming, utility and broad access of applications are vital. The development of smartphone applications is key to deliver the DM phenomenon to facilitate communication and engagement between clinicians and patients. A key element would be to personalise both treatments and applications using sensors and programming capabilities that would support significant benefits as summarised in Supplementary Table S3.

Evaluating the current DM landscape is equally important as developing novel applications. The accessibility of smartphones has given rise to multiple pilots of app-based longitudinal assessment programmes for chronic pain, which have shown promising early results (23, 24). Furthermore, the use of validated lifestyle devices such as the FitBit® as monitoring adjuncts could be combined with questionnaires and activity programmes to allow regular functional reassessment among chronic pain patients (25).

Therefore, the primary aims of this study were to: (1) identify and report the current prevalence of DM application in pain medicine; (2) identify and report the current DM application use within pain medicine. In this publication, we have explored the types of assessments, their use and deployment-related to DM applications.

## Materials and methods

An evidence synthesis methodology was developed for the purpose of this study, with a systematic review protocol published on PROSPERO (CRD42021248232). The Preferred Reporting Items for Systematic Reviews and Meta-analyses (PRISMA) was used to report findings.

### Search strategy and study selection

PubMed and ScienceDirect were used to identify relevant studies that were peer-reviewed and published in English between the 1st of January 1990 and 1st of January 2021. Search terms used included *Chronic Pain*, *Pain Clinical Trials*, *Pain medicine*, *Pain medicine clinical research* and *Digital Clinical Trials*. All studies using DM applications for chronic pain conditions were included. Only studies that were peer-reviewed and published in English were included. Suitable publications were selected using the PICO (Population/Participants, Intervention(s), Comparison, Outcome) strategy. An independent reviewer screened studies included within the study by reading the full text. Initial title and abstracts for identified articles were screened by 4 investigators. Inclusion and exclusion criteria were assessed against each study. This was followed with the screening of the full study article independently by 2 investigators and included into the final data pool.

### Data extraction and synthesis

**Figure 3:**
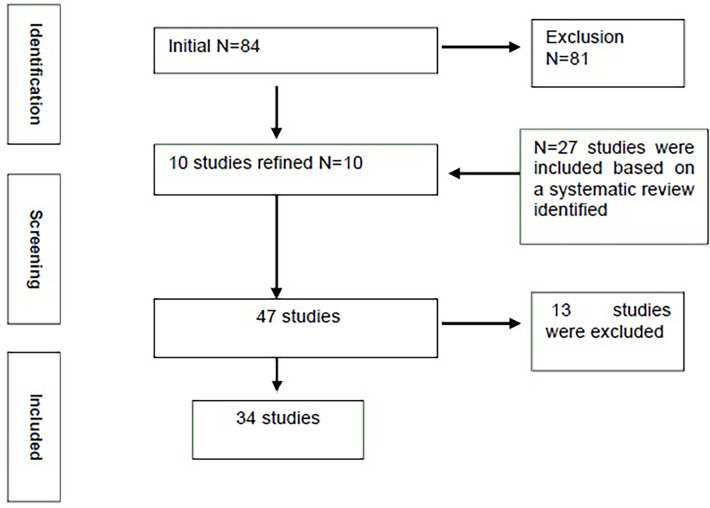
Representation of the PRISMA flowchart.

The data extraction process involved reading titles and abstracts followed by the application of the refinement protocol where the full text was reviewed and subsequently verified. Key study details such as study title, citation details, methods, findings, limitations, characteristics of the study and conclusions were extracted. Differing opinions were resolved by review and discussion between the lead authors. The authors remained unblinded regarding the publisher details. A full methodological description is demonstrated within the supplementary document (Figure 3).

### Data analysis

As all studies reported the mean and SD at several time points, a mathematical model was formulated as demonstrated in Supplementary Figure S1.

BPI (Brief Pain Inventory), NRS (Numeric Pain Rating Scale), PCP-S(Profile of Chronic Pain: Screen) and pain evaluation questionnaires were used to assess pain intensity and pain interference; HADS (Hospital Anxiety and Depression Scale), CES-D (Centre for Epidemiological Studies Depression), BDI (Beck's Depression Inventory), PHQ-9 (Patient Health Questionnaire-9), DASS (Depression Anxiety Stress Scales), GAD-7 (Generalised Anxiety Disorder-7) and STAI (State-Trait-Anxiety-Inventory) were used to assess depression and anxiety; FSS (Fatigue Severity Scale) and MOS (Medical Outcomes Study) sleep scale were used to assess fatigue and sleep. All studies reported the mean and SD of the questionnaires across several timepoints, at baseline and follow-up. The baseline questionnaire score was subtracted from the follow-up questionnaire score to standardize the data and remove the initial effect. Score changes between these two time points reflect the treatment effect. $\left(x_{e}^{0}-x_{e}^{1}\right)$ represented the change in the questionnaire scores between baseline (^0^) and follow-up (^1^) in the treatment group, which also indicated an improvement of treatments, and $\left(x_{c}^{0}-x_{c}^{1}\right)$ represented the change in the questionnaire scores between baseline (^0^) and follow-up (^1^) in the control group.

Therefore, $\left(x_{e}^{0}-x_{e}^{1})-(x_{c}^{0}-x_{c}^{1}\right)$ showed the mean difference (MD) of the change of score between the two groups, which is the outcome of focus. If $\left(x_{e}^{0}-x_{e}^{1})-(x_{c}^{0}-x_{c}^{1}\right)$ is positive, it indicates the treatment was beneficial for patients in improving symptoms of pain. However, if $\left(x_{e}^{0}-x_{e}^{1})-(x_{c}^{0}-x_{c}^{1}\right)$ is negative, it indicates the treatment had no effect on improving pain.

$$\mathrm{MD}=(x_{e}^{0}-x_{e}^{1})-(x_{c}^{0}-x_{c}^{1})$$

$$\mathrm{MD}∼N\left((m_{e}^{0}-m_{e}^{1})-(m_{c}^{0}-m_{c}^{1}),\;\frac{{s_{e}^{0}}^{2}}{n_{e}^{0}}+\frac{{s_{e}^{1}}^{2}}{n_{e}^{1}}+\frac{{s_{c}^{0}}^{2}}{n_{c}^{0}}+\frac{{s_{c}^{1}}^{2}}{n_{c}^{1}}\right)$$

The scales of the questionnaires were different, therefore standardized mean differences (SMD) were used to illustrate the change in the mean score of the treatment group vs. the control group from baseline to follow-up. The traditional form of SMD was

$$\hat{g_{k}}=\left(1-\frac{3}{4n_{k}-9}\right)\frac{\hat{u_{ek}}-\hat{u_{ck}}}{\sqrt{\left((n_{ek}-1)s_{ek}^{2}+(n_{ck}-1)s_{ck}^{2})/(n_{k}-2\right)}}$$

$$\hat{Var}(\hat{g_{k}})=\frac{n_{k}}{n_{ek}\cdot n_{ck}}+\frac{{\hat{g_{k}}}^{2}}{2(n_{k}-3.94)}$$

where $n_{k}=n_{ek}+n_{ck}$, $n_{ek}$, $\;\;\hat{u_{ek}}$, $s_{ek}$ are the number, mean and standard variation of treatment group. $n_{ck}$, $\;\;\hat{u_{ck}}$, $s_{ck}$ are the number, mean and standard variation of the control group. The 95% confidence interval (CI) was obtained by

$$\left(\hat{g_{k}})\pm 1.96*S.E.(\hat{g_{k}}\right)$$

where $S.E.(\hat{g_{k}})=\sqrt{\hat{Var}(\hat{g_{k}})}$.

$\hat{g_{k}}$ was transformed according to the traditional form, and $\hat{g_{k}}$ and $S.E.(\hat{g_{k}})$ were calculated for each study, with a random effect model used to pool the estimators. Funnel plot graphs demonstrated the publication bias. Subgroup analysis and *I*^2^ were used to explain heterogeneity and Egger's test was used to detect publication bias. All procedures were finished with STATA 16.1.

### Risk of bias

The risk of bias (RoB) table (Table 1) has been used to demonstrate the risk of bias within the randomised controlled trials used in the systematic review and meta-analysis. The RoB is reflective of a fixed set of biases within domains of study design, conduct and reporting. This combined with the quality check allows the findings of the study to be scientifically justified, and clinically viable.

**Table 1:** Risk of bias, according to the revised risk-of-bias tool for randomised trials (RoB 2.0) (67).

Author	Randomisation process	Deviations from the intended interventions	Missing Outcome Data	Measurement of the Outcome	Selection of the reported result	Overall
Bossen et al. (2013)	Some concerns^a^	Low risk	Low risk	Low risk	Low risk	Some concerns
Hedman-Lagerlöf, et al. (2018)	Low risk	Low risk	Low risk	Low risk	Low risk	Low risk
Krein et al. (2013)	Low risk	Low risk	Low risk	Low risk	Low risk	Low risk
Rini et al. (2015)	Low risk	Low risk	Low risk	Low risk	Low risk	Low risk
Williams et al. (2010)	Low risk	Low risk	Low risk	Low risk	Low risk	Low risk
Wilson et al. (2015)	Low risk	Low risk	Low risk	Low risk	Low risk	Low risk
Raj et al. (2017)	Low risk	Low risk	Low risk	Low risk	Low risk	Low risk
Guillory et al. (2015)	Low risk	Low risk	Low risk	Low risk	Low risk	Low risk
Berman et al. (2009)	Low risk	Low risk	Low risk	Low risk	Low risk	Low risk
Carpenter et al. (2012)	Low risk	Low risk	Low risk	Low risk	Low risk	Low risk
Menga et al. (2014)	Low risk	Low risk	Low risk	Low risk	Low risk	Low risk
O'moore et al. (2018)	Low risk	Low risk	Low risk	Low risk	Low risk	Low risk
Gentili et al. (2020)	Low risk	Low risk	Low risk	Low risk	Low risk	Low risk
Minen et al. (2019)	High risk^b^	Low risk	Low risk	Low risk	Low risk	High risk
Toelle et al. (2019)	Some concerns^c^	Low risk	Low risk	Low risk	Low risk	Some concerns
Blödt et al. (2018)	Low risk	Low risk	Low risk	Low risk	Low risk	Low risk
Irvine et al. (2015)	Low risk	Low risk	Low risk	Low risk	Low risk	Low risk
Schatz et al. (2015)	Low risk	Low risk	Low risk	Low risk	Low risk	Low risk
Nebojsa et al. (2017)	Low risk	Low risk	Low risk	Low risk	Low risk	Low risk
Sun et al. (2017)	Low risk	Low risk	Low risk	Low risk	Low risk	Low risk
Guétin et al. (2016)	Low risk	Low risk	Low risk	Low risk	Low risk	Low risk
Jamison et al. (2017)	Low risk	Low risk	Low risk	Low risk	Low risk	Low risk
Jibb et al. (2017)	Low risk	Low risk	Low risk	Low risk	Low risk	Low risk
Lee et al. (2017)	High risk^b^	Low risk	Low risk	Low risk	Low risk	High risk
Oldenmenger et al. (2016)	Low risk	Low risk	Low risk	Low risk	Low risk	Low risk
Huber et al. (2017)	Low risk	Low risk	Low risk	Low risk	Low risk	Low risk
Calner et al. (2017)	Low risk	Low risk	Low risk	Low risk	Low risk	Low risk Low risk
Chiauzzi et al. (2010)	Low risk	Low risk	Low risk	Low risk	Low risk	Low risk
Davis et al. (2013)	Low risk	Low risk	Low risk	Low risk	Low risk	Low risk
Dowd et al. (2015)	Low risk	Low risk	Low risk	Low risk	Low risk	Low risk
Lin et al. (2020)	Low risk	Low risk	Low risk	Low risk	Low risk	Low risk
Nordin et al. (2016)	Low risk	Low risk	Low risk	Low risk	Low risk	Low risk
Ruehlmana et al. (2012)	Low risk	Low risk	Low risk	Low risk	Low risk	Low risk
Ström et al. (2000)	Low risk	Low risk	Low risk	Low risk	Low risk	Low risk
Anderson et al. (2004)	Low risk	Low risk	Low risk	Low risk	Low risk	Low risk
Lovell et al. (2010)	Low risk	Low risk	Low risk	Low risk	Low risk	Low risk
Guétin et al. (2018)	Low risk	Low risk	Low risk	Low risk	Low risk	Low risk
Oldenmenger et al. (2018)	High risk^b^	Low risk	Low risk	Low risk	Low risk	High risk

^a^ Some concerns due to missing information regarding the allocation concealment.

^b^ High risk because of lack of randomisation.

^c^ Some concerns due to deviation from the protocol resulting in a 53:48 distribution of participants.

**Figure 4:**
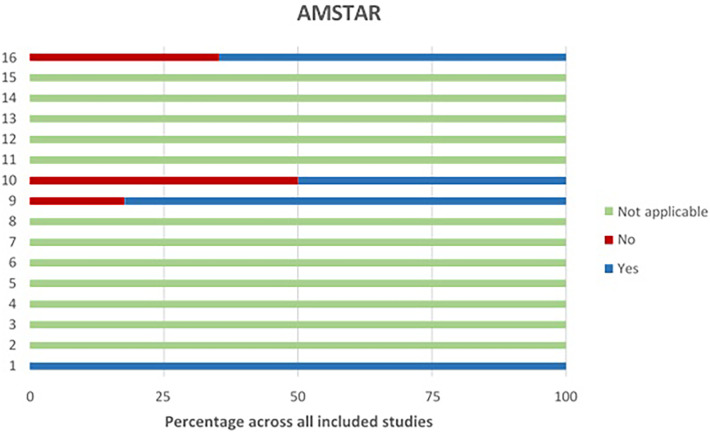
AMSTAR assessment.

AMSTAR (68) was used also to assess methodological quality, where the total scores range from 0 to 11 (see Figure 4, below). An article would be considered as good quality with a score of 8–11, moderate 4–7 and low 0–3.

### Outcomes

Outcomes of this study were reported *via* the meta-analysis which was based on the availability of statistics reported by the systematically included studies. The following are the outcomes of this study:

- Prevalence of DM applications, including categories
- Prevalence of chronic pain conditions using DM applications for self-reporting purposes
- Standard Mean Difference of pain outcomes of depression, anxiety, pain inferences, and fatigue and sleep problems between DM applications and non-DM routine care
- Clinical significance of the prevalence data
- Research significance of the prevalence data
- Critical interpretation of the identified data
- Common themes identified within the prevalence data

## Results

The search yielded 84 publications, with 38 (23, 26–62) included as part of the systematic review (Table 2). Of the 38 studies, 7 were cross-sectional and lacked a control group. Eight studies comprised of a control and treatment group, although they either lacked statistical information completely or inconsistencies were identified that were associated with the mean and SD at baseline and beta coefficients at follow-up timepoints. Therefore, 16 (35, 38, 39, 41, 43, 45–50, 53, 56, 60–62) were excluded and 22 (23, 26–34, 36, 37, 40, 42, 46, 51, 52, 54, 55, 57–59) were included into the final meta-analysis (Table 3).

**Table 2:** Characteristics of the systematically included studies.

Author	Diagnosis/Treatment method	Digital application and method of application delivery	Study type	Sample size	Country	Exposure
Bossen et al. (2013)	Intervention	Web-based intervention	RCT	199	Netherlands	Osteoarthritis pain
Hedman-Lagerlöf, et al. (2018)	Intervention	Web-based intervention	RCT	140	Sweden	Fibromyalgia
Krein et al. (2013)	Intervention	Web-based intervention	RCT	229	United States	Chronic low back pain
Rini et al. (2015)	Intervention	Web-based intervention	RCT	113	United States	Osteoarthritis pain
Williams et al. (2010)	Intervention	Web-based intervention	RCT	118	United States	Fibromyalgia
Wilson et al. (2015)	Intervention	Web-based intervention	RCT	92	United States	Chronic noncancer pain
Raj et al. (2017)	Intervention	Web-based intervention	RCT	214	Norway	Cancerrelated pain
Guillory et al. (2015)	Chatbots	Text message and mobile app	RCT Feasibility	68	United States	Chronic noncancer pain
Berman et al. (2009)	Chatbots	Web-based intervention	RCT	78	United States	Chronic pain
Carpenter et al. (2012)	Chatbots- Cognitive behavioral therapy with chapters	Web-based intervention	RCT Pilot	141	United States	Chronic low back pain
Menga et al. (2014)	Chatbots- Cognitive behavioral therapy with chapters	Web-based intervention	RCT	44	United States	Fibromyalgia
O'moore et al. (2018)	Chatbots- Cognitive behavioral therapy with chapters	Web-based intervention	RCT	69	United States	Osteoarthritis pain
Gentili et al. (2020)	Mobile app based acceptance therapy	Mobile based intervention	RCT pilot	31	Sweden	Chronic pain
Minen et al. (2019)	Mobile app based behavioral therapy	Mobile based intervention	Crosssectional - Feasibility	51	United States	Migraine
Toelle et al. (2019)	Mobile app based therapy	Mobile based intervention	RCT	94	Germany	Chronic nonspecific low back pain
Blödt et al. (2018)	Mobile app based self-acupressure	Mobile based intervention	RCT - Pragmatic	221	Germany	Menstrual pain
Irvine et al. (2015)	Mobile app based self-management	Mobile based intervention	RCT	597	United States	Chronic low back pain
Schatz et al. (2015)	Mobile app based coping, pain and activity	Mobile based intervention	RCT	46	United States	Chronic pain for paediatric sickle cell
Nebojsa et al. (2017)	Mobile app and an wearable activity monitor	Mobile based intervention	RCT	211	United States	Osteoarthritis pain
Sun et al. (2017)	Mobile app for pain management	Mobile based intervention	RCT	46	China	Cancer-related pain
Guétin et al. (2016)	Mobile app delivering music therapy for pain	Mobile based intervention	RCT	106	France	Chronic pain
Jamison et al. (2017)	Mobile app based daily assessment and treatment	Mobile based intervention	RCT - pilot	90	United States	Chronic pain
Jibb et al. (2017)	Mobile apps	Mobile based intervention	RCT -pragmatic	40	Canada	Cancer-related chronic pain among the adolescent
Lee et al. (2017)	Mobile app-based exercise program	Mobile based intervention	Crosssection single group repeated measure	23	Korea	Neck pain
Oldenmenger et al. (2016)	Mobile apps	Web-based intervention	quantitative	48	Netherlands	Cancer-related pain
Huber et al. (2017)	Mobile app and EHR*	Mobile based intervention	Retrospective RCT*	180	Germany	Chronic low back pain
Calner et al. (2017)	Intervention	Web-based intervention	RCT	109	Sweden	Musculoskeletal pain
Chiauzzi et al. (2010)	Intervention - self-management	Web-based intervention	RCT	199	United States	Chronic pain
Davis et al. (2013)	Intervention of mindfulness	Web-based intervention	RCT	79	United States	Fibromyalgia
Dowd et al. (2015)	Online mindfulness-based cognitive therapy intervention	Web-based intervention	RCT	124	Ireland	Chronic pain
Lin et al. (2020)	Mobile apps	Web-based intervention	RCT	302	Germany	Multimodal pain
Nordin et al. (2016)	Intervention for web behaviour change	Web-based intervention	RCT	109	Sweden	Multimodal pain
Ruehlmana et al. (2012)	Intervention self-management	Web-based intervention	RCT	305	United States	Chronic pain
Ström et al. (2000)	Intervention – self-management	Web-based intervention	RCT	45	Sweden	Recurrent headache
Anderson et al. (2004)	Intervention - video and booklet	Web-based intervention	RCT	97	United States	Cancer-related pain
Lovell et al. (2010)	Intervention – video and booklet	Web-based intervention	RCT	217	Australia	Cancer-related pain
Guétin et al. (2018)	Smartphone-based intervention	Mobile-based intervention	RCT	62	France	Chronic painful conditions
Oldenmenger et al. (2018)	Intervention – internet applications	Web-based intervention	cohort study	84	Netherlands	Cancer-related pain

* EHR, electronic health records; RCT, randomised clinical trial.

**Table 3:** Studies included within the meta-analysis.

Study ID	Author	Digital applications	Study type	Sample size	Country	Exposure	*p*-value
1	Bossen et al. (2013)	Web-application	RCT	199	Netherlands	Osteoarthritis pain	0.33 (pain intensity); 0.09 (depression); 0.007 (anxiety)
2	Hedman-Lagerlöf et al. (2018)	Web-application	RCT	140	Sweden	Fibromyalgia	<0.001 (depression); <0.001 (anxiety); <0.001 (fatigue)
3	Krein et al. (2013)	Web-applications	RCT	229	United States	Chronic low back pain	Not provided
4	Rini et al. (2015)	Web-application	RCT	113	United States	Osteoarthritis pain	Not provided
5	Williams et al. (2010)	Web-application	RCT	118	United States	Fibromyalgia	Not provided
6	Wilson et al. (2015)	Web-application	RCT	92	United States	Chronic noncancer pain	0.22 (pain intensity); 0.25 (depression)
7	Raj et al. (2017)	Web-application	RCT	214	Norway	Cancer-related pain	Not provided
8	Guillory et al. (2015)	Chatbots	RCT feasibility	68	United States	Cancer-related pain	Not provided
9	Berman et al. (2019)	Chatbots	RCT	78	United States	Chronic pain	Not provided
10	Menga et al. (2014)	Chatbots	RCT	44	United States	Fibromyalgia	0.005 (severity of fibromyalgia)
11	O'moore et al. (2018)	Chatbots	RCT	69	Australia	Osteoarthritis pain	Not provided
12	Gentili et al. (2020)	Mobile apps	RCT	94	Germany	Chronic low back pain	0.021 (pain intensity)
13	Blödt et al. (2018)	Mobile apps	RCT	221	Germany	Menstrual pain	0.026 (pain intensity)
14	Schatz et al. (2015)	Mobile apps	RCT	46	United States	Chronic pain	0.1 (negative affect)
15	Sun et al. (2017)	Mobile apps	RCT	46	China	Cancer-related pain	<0.01 (pain intensity)
16	Calner et al. (2017)	Mobile apps	RCT	109	United States	Musculoskeletal pain	0.37 (intensity)
17	Chiauzzi et al. (2010)	Mobile apps	RCT	199	United States	Chronic pain	Not provided
18	Dowd et al. (2015)	Mobile apps	RCT	124	Ireland	Chronic pain	Not provided
19	Lin et al. (2020)	Mobile apps	RCT	302	Germany	Multimodal pain	0.01 (pain intensity); <0.01 (depression); 0.44 (anxiety); <0.01 (pain interference)
20	Ruehlmana et al. (2012)	Mobile apps	RCT	305	United States	Chronic pain	0.2 (pain intensity); 0.06 (depression); 0.15 (anxiety); 0.3 (pain interference)
21	Ström et al. (2000)	Mobile apps	RCT	45	Sweden	Recurrent headache	Not provided
22	Anderson et al. (2004)	Web-application	RCT	84	Netherlands	Cancer-related pain	Not provided

### Meta-analysis

All 22 studies included in the meta-analysis reported more than one pain-related symptom. One primary outcome reported in 15 studies was pain intensity. 11 reported depressive symptoms and 9 anxiety symptoms. Pain interference was reported by 4 studies. Fatigue and sleep problems were included as secondary outcomes in two and one study respectively. Meta-analyses were conducted for each outcome separately.

### Pain intensity

**Figure 5:**
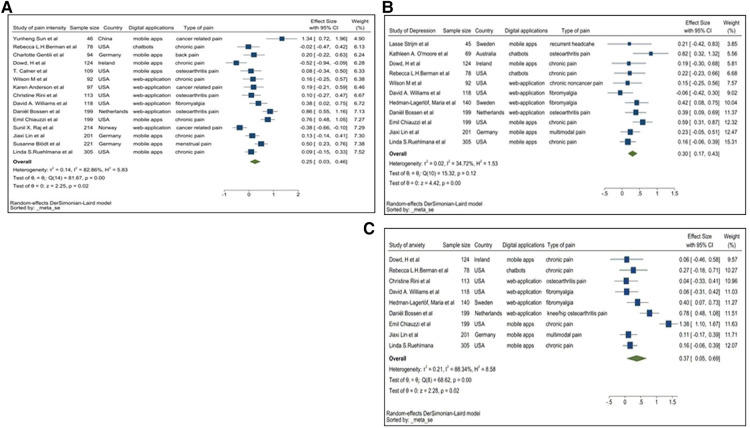
(**A**) Forest plot of pain intensity. (**B**) Forest plot of depression. (**C**) Forest plot for anxiety.

All 15 studies provided the mean and SD. Therefore, the meta-analysis was based on the mean and SD. Figure 5 demonstrates a pooled SMD of 0.25 with a 95%CI of 0.03–0.46. SMD is statistically higher than 0; therefore, pain scores within the treatment group reduced compared to the control group, suggesting DM applications can significantly reduce symptoms of pain. A high heterogeneity of *I*^2^ = 82.86% was identified for this group (*p *= 0.02). A subgroup analysis was conducted to analyse the possible source of heterogeneity.

### Depression

The 11 studies reporting depressive symptoms used various assessment tools, including the Centre for Epidemiological Studies-Depression (CES-D), Beck Depression Inventory (BDI), Patient Health Questionnaire-8 and −9 (PHQ-8, PHQ-9), Hospital Anxiety and Depression Scale (HADS) and the Depression Anxiety Stress Scales (DASS). Ruehlman and colleagues (2012) used CES-D and DASS to assess the depression of the participants twice. To avoid duplication we used only one (CES-D) of the means and SD of these two assessments so that 11 studies were included in meta-analysis. Figure 7 showed that the pooled SMD was 0.30 with a 95%CI of 0.17–0.43, suggesting the use of DM applications reduced depression symptoms compared with the usual standard care, with an elevated heterogeneity of *I*^2^ = 34.72% (*p *= 0.00).

**Figure 6:**
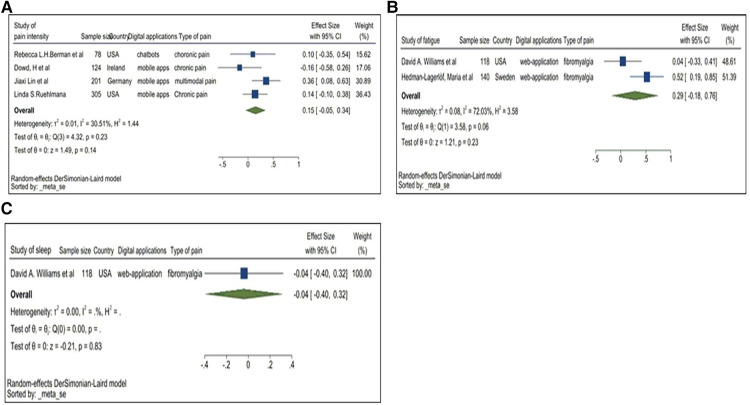
(**A**) Forest plot for pain interference. (**B**) Forest plot of fatigue. (**C**) Forrest plot of sleep.

**Figure 7:**
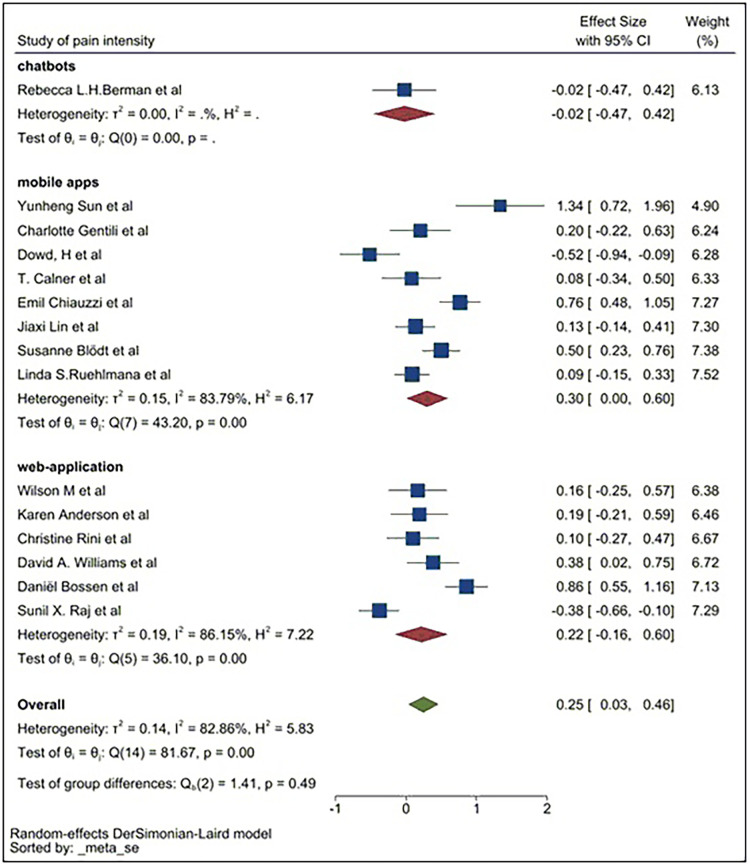
Subgroup analysis for pain intensity (web-application, mobile apps, chatbots).

### Anxiety

**Figure 8:**
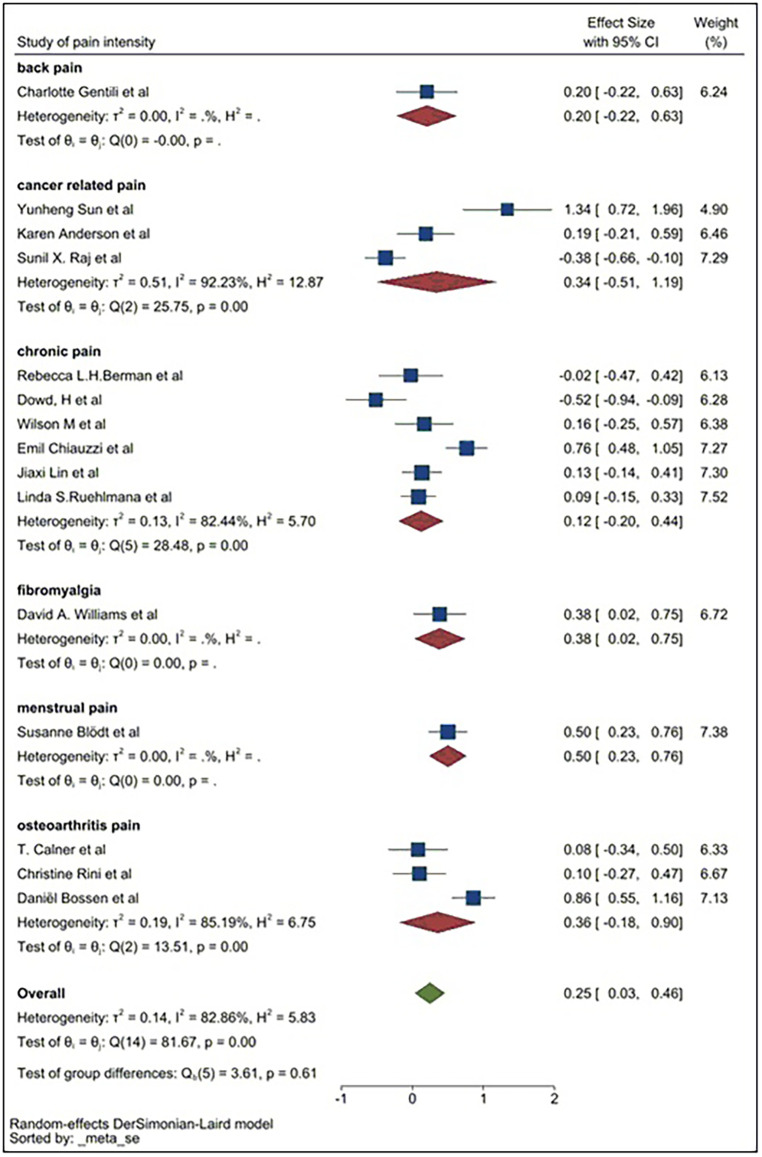
Subgroup analysis for pain intensity (by pain type).

Within the 9 studies reporting anxiety as a clinical outcome among chronic pain participants, the pooled SMD was 0.37 with a 95%CI of 0.05–0.69 (Figure 8). The SMD is significantly greater than 0, indicating anxiety symptoms among participants following use of DM applications improved more than the control group. Additionally, a treatment effect greater than 0 was seen in each individual study, thus each study concluded that DM applications improve anxiety symptoms compared with controls. Heterogeneity seen within this dataset was high with *I*^2^ = 88.34% (*p *= 0.02), indicating our factors such as sample size, country of subjects, the type of digital applications used and type of pain influence the conclusion of meta-analysis. Due to the number of studies is too small, it's hard to conduct subgroup analysis or meta-regression here. To obtain more precise and convincing conclusion, more studies are needed here.

### Pain interference

Four studies reported pain interference, an important outcome in pain research. Figure 6A demonstrates a pooled SMD of 0.15 with a 95%CI of −0.05 to 0.34. SMD was not significantly higher than 0, suggesting that the improvement within the treatment group was not significantly greater than control group. DM applications appear to have no effect on participants exposed to the application indicating mild heterogeneity. Therefore, a lack of a statistically obvious effect has been observed within the pooled dataset.

### Fatigue/sleep

Two studies reported on fatigue and one study on sleep issues. The forest plots for these factors are illustrated below (Figures 6B,C).

The pooled SMD for fatigue was 0.29, indicating the treatment group improved following the completion of the DM application use. However, this conclusion is not statistically significant given the 95%CI of −0.18 to 0.76. This could be due to the presence of only 2 studies, and more is needed to reach a conclusion.

The pooled SMD for sleep issues was −0.04 with a 95%CI of −0.4 to 0.32. This indicates that DM applications did not improve sleep-related issues and is of a lower score compared to the control group. However, to provide a more comprehensive conclusion to this phenomenon, further studies would be required.

### Subgroup analysis

Subgroup analysis was conducted to identify the source of raised heterogeneity when considering studies reporting on pain intensity. Initial analysis considered the categories of DM applications which included web-applications, mobile apps and chatbots. The analysis is demonstrated in Figure 7.

The pooled SMD for web-applications was 0.22, indicating web-applications could reduce the intensity of pain compared to the control group. The pooled SMD of mobile apps was 0.30. This demonstrates a larger effect size in relieving the intensity of pain compared to control groups and to those using web-applications. The pooled SMD for chatbots was −0.02, indicating chatbots have a limited effect in reducing the intensity of pain in patients compared to the controls. Heterogeneity remained high in all three subgroups, so a second subgroup analysis was conducted based on exposure of pain symptoms. The pain exposure sub-group analysis included identified specific pain conditions: fibromyalgia, back pain, chronic pain, osteoarthritis pain, menstrual pain, and cancer-related pain (Figure 8).

**Figure 9:**
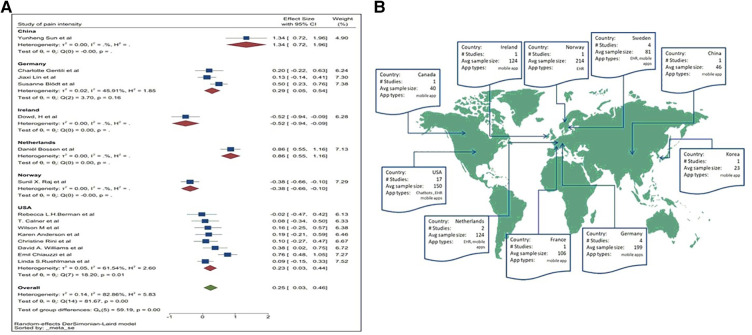
(**A**) Subgroup analysis for pain intensity (by country). (**B**) Geographical spread of data collected for the systematic review.

Heterogeneity could only be calculated in three of the subgroups. It remained high within these pooled subgroups, although at a lower level compared to previous analyses. Cancer-related pain reported the highest level of heterogeneity (*I*^2 ^= 92.23%). Chronic pain and osteoarthritis pain groups reported an *I*^2^ of 82.44% and 85.19% respectively. However, due to the limited number of studies, pain and digital application exposures, the effect size could not be comprehensively assessed. A third subgroup analysis was conducted based on geographical locations (Figures 9A,B).

A sub-group analysis by country found that DM applications appear to be effective within populations in America, China, Germany, and Netherlands, while for Ireland and Norway, a statistically significant effect was lacking. Only mild heterogeneity levels were indicated for America (*I*^2 ^= 61.54%) and Germany (*I*^2 ^= 45.91%). The heterogeneity may well be due to nationality and ethnicity.

### Sensitivity analysis

**Figure 10:**
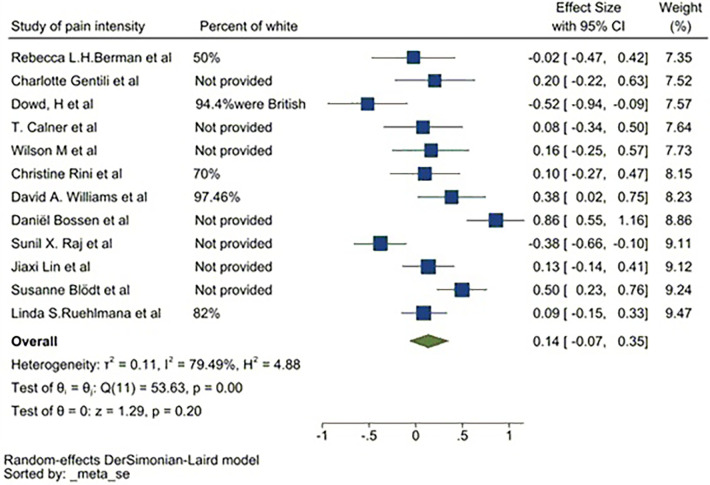
Sensitivity analysis without 3 BAME studies (29, 37, 56).

Based on the meta-analysis and the sub-group analysis conducted to demonstrate pain intensity outcomes from the digital tools reported by Anderson et al. (59), Chiauzzi et al. (52) and Sun et al. (44), the standard mean deviation (SMD) was high. The primary populations of Chiauzzi and colleagues (2010) and Anderson and colleagues (2004) were African American followed by Hispanic, whilst Sun et al. (2017) reported on a population of Chinese patients. Similar ethnicity and race patterns were found among 12 of the 15 studies in the meta-analysis. Of these, 5 reported ethnicity, although over 50% of the sample size was Caucasian. The other 7 did not provide specific percentages of the Caucasian representation. A sensitivity analysis was conducted to assess ethnic variability within the pooled sample size, which resulted in a SMD of 0.14 with a 95%CI of −0.07 to 0.35 (Figure 10).

The sensitivity analysis reveals DM applications appear to benefit patients. However, to conclusively demonstrate a statistical significance more studies would be required. The *p*-value where the reported SMD was greater than 0 was 0.2, indicating there is a 90% probability that the DM application would have a positive impact on the patient's pain. It is equally vital to recognize that the predominantly African American and Hispanic population-based studies reported a SMD of 0.76 with a 95% of 0.48–1.05, and a study consisting entirely of African American and Hispanic participants reported a SMD of 0.19 (95%CI −0.21 to 0.59). Sun et al. (2017) reported a SMD of 1.34 with a 95% CI of 0.72–1.96. Therefore, DM applications appear to have a positive impact on patients.

**Figure 11:**
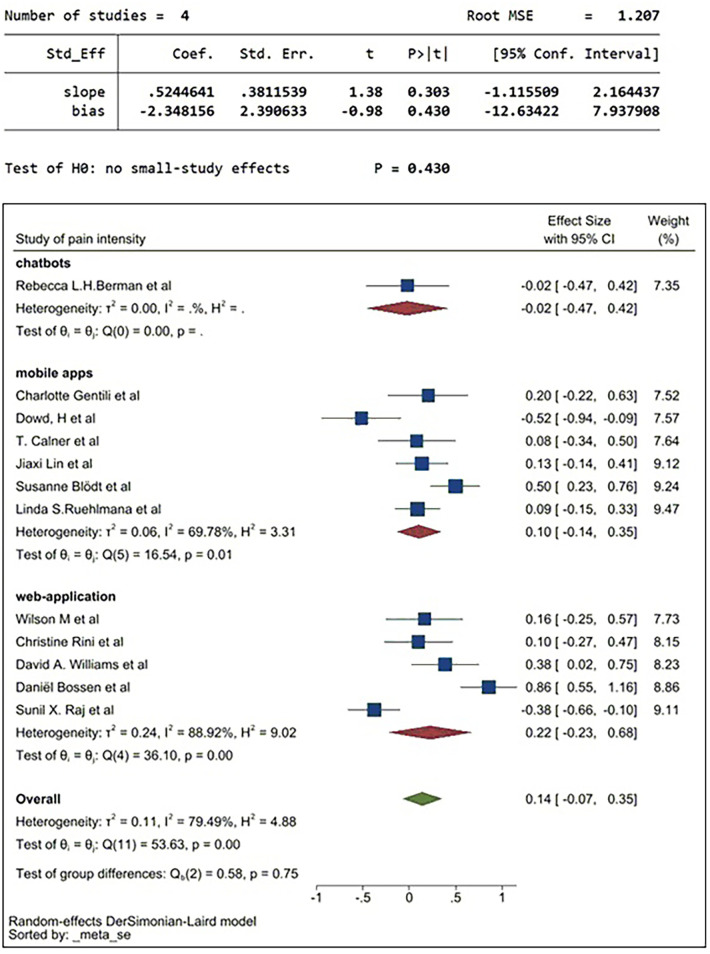
Sensitivity analysis without 3 BAME studies (29, 37, 56) (sub-grouped by digital applications).

The sensitivity analysis shown in Figure 11 demonstrates strong heterogeneity. The main source of heterogeneity could be the difference in the treatment interventions deployed by way of the DM application. As this is associated with the interventions themselves rather than the DM applications, it is beyond the scope of this study, and could be explored in the future. Pooled SMD of web-application, mobile apps and chatbots were 0.22, 0.1 and −0.02 respectively. Figure 11 demonstrates that the most effective DM application could be mobile apps since web-applications are not a self-reported method.

### Publication bias

**Figure 12:**
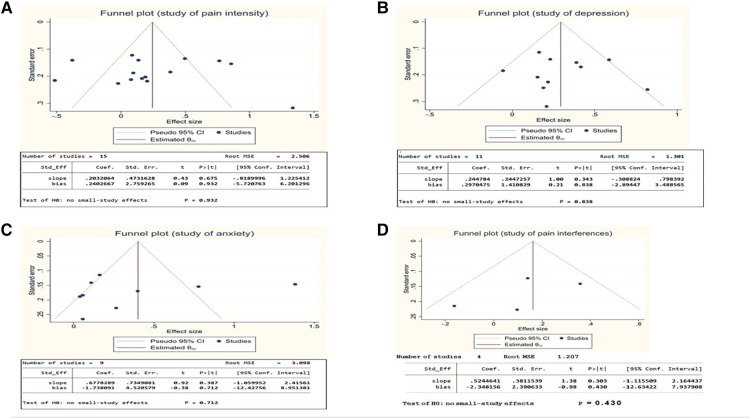
(**A**) Funnel plot for pain intensity & Egger's test for pain intensity. (**B**) Funnel plot for depression & Egger's test for depression. (**C**) Funnel plot for anxiety & Egger's test for anxiety. (**D**) Funnel plot for pain inferences & Egger's test for pain inferences.

Publication bias was assessed and reported using funnel plots and Egger's test to examine the small-study effect. Publication bias appears to be smaller among studies associated with fatigue and sleep, and higher in studies demonstrating pain intensity, depression, anxiety, and pain interferences. There is a lack of significant publication bias based on the funnel plot (Figure 12A, below). The Egger's test *p*-value is 0.932, indicating the lack of a small study effect. However, there are 5 studies that fell outside the 95%CI which could affect our detection of publication bias. The *p*-value is not high but it is limited by the data and experimental quality.

Figures 12B,C indicate a lack of publication bias statistically for depression and anxiety, with Egger's test *p*-values of 0.838 and 0.712 respectively. Pain inferences (Figure 12D), which was included in four studies, had an Egger's test *p*-value of 0.43, which demonstrates we cannot detect a publication bias. The low numbers of studies reporting outcomes for fatigue and sleep problems meant analysis of publication bias was not possible.

## Discussion

The prevalence of DM applications within pain research appear to be moderate and is focused around developed countries such as America and Germany. China appears to be the only country within Asia to have conducted a study to assess the use of DM applications among patients with chronic pain. This demonstrates an urgent need to conduct evaluations of these DM applications in low-income and middle-income countries to optimise and evaluate the efficacy and acceptability among patients and clinicians. Patient-reported DM applications identified in the systematic review could be categorised primarily as mobile apps and chatbots, as EHR systems were used to assess pain-associated outcomes. As a result of these differences, the prevalence of DM applications was meta-analysed at a granular level to identify and report pain outcomes such as depression, anxiety, pain intensity and pain inference that were assessed by the tools. The lack of uniformity among the assessments used within the applications are another issue that requires further elaboration if these are to be used by clinicians as part of a patient's ongoing clinical management. The assessments used also appear to be non-specific to a particular group of patients. Often, the studies did not report on underlying conditions or if the pain conditions had a clinical diagnosis. Thus, it is challenging to demonstrate that users demonstrated true clinical benefit. This suggests there is little quantifiable data to provide a comprehensive conclusion in terms of the generalisability and feasibility of these applications globally.

We identified multiple themes and sub-themes in this analysis that were pooled as mobile applications, EHR and chatbots. Mobile applications have grown rapidly to support the management of pain disorders such as migraine, back pain and fibromyalgia by offering educational components, exercise platforms, relaxation techniques and mindfulness-based options to name a few. These options provide feedback and allow engagement and adherence of the users. This may explain why mobile applications demonstrated better results compared with other DM applications in the management of chronic pain. Another facet to consider is the inclusion of these datasets to maintain a structured approach to deliver effective continuity of care provision. Trials promoting the evaluation of data in a comprehensive manner through systems that allow the standardisation and acceptance of quality data would increase the acceptance of digitised data. Trials involving DM applications that incorporate AI-based clinical algorithms to assist with the evaluation of pain and outcomes in patients with cancer appear encouraging (63).

Ledel Solem and colleagues (2019) reported adult participants were in favour of using DM-based self-management interventions for chronic pain management (64). Patients felt that the accessibility, usability, and personalisation were vital for DM tools, and suggested that these should be further developed to distract them from pain, regardless of pain intensity and cognitive capacity.

The benefits of harnessing DM within the context of pain medicine could improve both clinical and patient-reported outcomes. Evidence-gathering to support therapeutic efficacy for pharmacological or surgical treatments requires effective and robust methodology, yet rigid traditional trial designs remain inefficient and struggle with implementation into clinical practice, limiting sustainability within healthcare systems. Computer-based technology could address these obstacles in research. The flexibility and accessibility of digital technology enables a more convenient and improved consenting process. This could allow easier enrolment and participation in studies for populations disadvantaged by mobility or literacy issues. Increased recruitment and retention lead to larger study populations with greater data validity, and aids researchers by speeding up recruitment and assessment of large trial populations.

Digital clinical trials are becoming more poignant to test various complex and technological interventions, as well as a conducting follow-up of participants in large multi-center global clinical trials. Digital clinical trials are key in collating all the above factors, as it is a fundamental tool in assessing the efficacy and safety of novel drugs, medical devices, and health system interventions. In the era of COVID-19, digital clinical trials have proven to be highly effective and valuable for continuity of clinical research. Traditional clinical trials have demonstrated the validity, acceptability, and sustainability of the interventions, whilst digital clinical trials could leverage technologies to engage and report trial-specific measurements associated with the interventions being tested at a lower cost (63). Conceptualising digital clinical trials for pain medicine could have added benefits, especially for patients who could report pain episodes daily. That would allow digital analytics to assess considerations clinicians need to make when developing clinical treatments. Additionally, data science approaches could be leveraged in this instance to develop novel clinical methods to best utilise trial data with “real-world” data to develop aggregated datasets. These could be used to promote multi-morbid clinical research, which is vital in furthering clinical practices associated with pain medicine.

### Limitations

Unified approaches of conducting DM application assessments were lacking across all 3 categories identified and reported within the scope of this study. As a result, the pooled analysis conducted limits the generalizability of the findings. It is evident that the lack of validation in digital applications is another rate-limiting factor in furthering the use of these among clinical populations.

In terms of DM overall, the clinical databases through which they operate would been to be encrypted and backed up to ensure data reliability and protection from information loss (17, 18). The data stored in this system could be used to formulate medical decisions hence all data recorded and stored must be original and accurate (17, 18). It is often difficult to predict how the software will operate, especially in its early days, so it is vital that all patient information is safely stored; should the worst happed, their clinical data is not lost or damaged.

### Future research

Research papers and databases were used in this review. Whilst the findings are compelling, there is the absence of real-world clinical studies to further validate these findings. A study where a digital medicine software is used in a clinical setting would develop understandings in the applicability and feasibility of such technology. Comparison between control and experimental participant group would enable outcomes to be assessed for efficacy and outcome monitoring.

It would be insightful to see application to a wider variety of healthcare disciplines to understand the various data management processes that would go underway (18). This would enable a better understanding of how DM would operate, as well as highlighting any issues or important point that need to be noted. On another note, most research into using ML in healthcare settings had looked at supervised usage, where healthcare professional are monitoring machinery and results (18). The results from unsupervised ML processes would be interesting to see in order to determine the efficacy and reliability of such software (18).

## Conclusion

The pain medicine ecosystem has a plethora of research studies, although those in population research, prevention, clinical trials, and education, as well as training, need to evolve if improvements are to be made clinically. This could integrate evolving DM concepts, including AI applications, that could improve patient-reported outcomes. It is, therefore, important to conduct further well-designed digital clinical trials.

Another concern based on evidence ascertained in this study is the minimal use of clinical trials to test DM applications; therefore, the efficacy and efficiency of these, as well as the generalizability to a wider population, remain limited. Pragmatic and novel methods of conducting clinical trials would be beneficial in providing credible evidence before these DM applications are used within clinical practice. Alternatives such as simulation studies using *real-world* environments could be used to test novel DM applications, given the complexities around conducting pain research. Similarly, it may be beneficial for patients to gain access to DM applications more quickly, especially those managing chronic pain. Therefore, a paradox of “*no evidence, no implementation vs. no implementation, no evidence*” is a challenge to clinicians, patients, policymakers, and clinical researchers alike. Using simulation methods, where possible, could provide an alternative method to overcome this paradox, although there may be limitations that would need considering as it not always feasible to design precise simulations or perform competency validation. The proliferation of digital technologies would provide the leverage to optimise global care by way of mobile platforms, to open better avenues, and to measure outcome data from wearable devices. These applications use real-world data that could benefit patients and clinicians alike. Thus, the use of DM in pain medicine could promote a myriad of benefits.

## Data Availability

The original contributions presented in the study are included in the article/**Supplementary Material**, further inquiries can be directed to the corresponding author/s.
