# Supplementary material for: A systematic review and meta-analysis of digital application use in clinical research in pain medicine
Source: Front Digit Health. 2022 Nov 2;4:850601. doi: 10.3389/fdgth.2022.850601 (PMC9668017; doi:10.3389/fdgth.2022.850601)
Supplement: Supplementary file 1 [file Image1.pdf]

Supplementary Figure 1: Mathematical model for data analysis

$$x_e^0 \sim \mathcal{N}(m_e^0, {s_e^0}^2)$$

$$x_e^1 \sim \mathcal{N}(m_e^1, {s_e^1}^2)$$

$$x_c^0 \sim \mathcal{N}(m_c^0, {s_c^0}^2)$$

$$x_c^1 \sim \mathcal{N}(m_c^1, {s_c^1}^2)$$
