# Supplementary material for: A systematic review and meta-analysis of digital application use in clinical research in pain medicine
Source: Front Digit Health. 2022 Nov 2;4:850601. doi: 10.3389/fdgth.2022.850601 (PMC9668017; doi:10.3389/fdgth.2022.850601)
Supplement: Supplementary file 2 [file Datasheet1.pdf]

Supplementary Table 1. Key clinical features and classification of pain

**Categories of pain**	**Summary**	**Interventions-Drug**	**Interventions-Medical devices**
Nociceptive pain	Somatic or visceral pain arises from actual or threatened damage to nonneural tissue and is due to the activation of nociceptors.	Physical therapy, Medications, Injection, Treatment of the underlying cause, NSAID	Injection, Treatment of the underlying cause
Inflammatory pain	Cardinal feature of an inflammatory state of hypersensitivity where innocuous stimuli induce pain. This is regulated by prostaglandin receptors EPI1, EP2, EP3 and EP4.	Anti-inflammatory medicine such as Aspirin, Ibuprofen, Diclofenac, Flurbiprofen, Mefanamic acid, Nabumetone, Piroxicam and Naproxen	Laser therapy systems, electrotherapy device, non-invasive heat therapy, infrared light therapy, pulsed electromagnetic field therapy and electrode induced pain blocker
Dysfunctional/Functional Pain	Pain without obvious organic cause.	Physical Therapy, Cognitive Behavioural Therapy (CBT)	TENS, External Neuromodulation
Neuropathic pain	Pain caused by a lesion or disease of the somatosensory nervous system.	Anti-neuropathic medication	Neuromodulation, Nerve blocks

Supplementary Table 2. Description of Regulatory Guidelines by Organisation

**Organisation/Regulator**	**Data published**	**Guidelines/Regulations**	**Description**
MHRA (Medicines and Healthcare Products Regulatory Agency)	2020	Medical Device Regulations (MDR)	The medical device directive was amended to the MDR to accommodate artificial intelligence

			applications that provide medical advice or act as a clinician aid. In addition, the UK conformity assessed (UKCA) marking that is equivalent to CE marking. The UKCA marking is a standard required for goods placed on the market in Great Britain
Code of conduct for data-driven health and care technology	2019	A policy document with 10 principles to evaluate digital health solutions	There is an associated code of conduct within the United Kingdom
NICE (National Institute of Health and Care Excellence) evidence standards framework	2019	The evidence tiers are cumulative therefore, the framework aims to assess digital health technologies (DHT) from a specific risk and best practice perspective.	The framework demonstrates the standards required for the evidence developed or available about digital health technologies that may or may not fall within the remit of medical devices, including those associated with artificial intelligence. This framework includes evidence associated with effectiveness specific to the intended purposes and user. This also includes evidence associated with the economic impact affiliated with financial risk.
FDA (Food and Drug Administration)	2016	Benefit-risk framework for medical devices	A generic framework to evaluate medical

			devices risks and benefits. For example, this framework can assess dimensions such as risk severity and the likelihood of risk as well as false positive or false negative results.
WHO (World Health Organisation)	2016	WHO monitoring and evaluating digital health interventions	A generic framework to evaluate and validate digital solutions throughout the lifecycle of the innovation.
IMDRF (International Medical Device Regulation Forum)	2017	Multiple definitions are included and developed by the International Medical Device. Regulators to manage these across multiple countries	The scope of this framework is a foundational approach to address unique challenges which include common vocabulary to identify specific information to support healthcare decision making and/or healthcare conditions with core functions
MEDDEV 2.1 (Medical Device Regulations)	2016	Guidelines on the quantification and classification of standalone software with a medical purpose and are associated within the regulatory framework of medical devices. These aspects are part of the European Union	These guidelines describe any medical tool when they do not fall within the remit of software, or in vitro diagnostic devices or a medical device
FDA (Food and Drug Administration) precertification program	2019	This is a software precertification program developed as a pilot	This program includes a test plan designed to evaluate the

			Excellence Appraisal and Streamlined Review composites that would formulate quality assurance for the safety and effectiveness of the software prior to implementation clinically.
FDA (Food and Drug Administration)-SaMD (Proposed regulatory framework for modifications to artificial intelligence/machine learning such as software as a medical device)	2019	SaMD demonstrates a risk categorisation framework inclusive of a risk-based approach to categorise intended use	This framework assesses 2 key descriptions of intended use; state of the healthcare situation or clinical condition and the patient population it is intended for in terms of risk (critical; serious, or nonserious healthcare situation/condition; and the information provided by the SaMD to formulate the decision for the intended user to diagnose or treat or clinically manage the condition.
FDA (Food and Drug Administration) Medical mobile application guidance	2018	The purpose of this guidance is for mobile platform as defined as a commercial computer platform with or without wireless connectivity that could be hand handled such as a platform downloadable to a smart phone or tablet.	This guidelines document is relevant to assess mobile applications where it is a software that is tailored to a mobile platform. This also includes device software functions defined under section 201(h) of the FD&C Act which could be conformed as an

			accessory to an existing regulated medical device or to transform a mobile platform into a regulated medical device.

Supplementary Table 3. Summary table of current uses of digital applications within clinical medicine

**Benefits**	**Summary**	**Application examples**
Improved access	Use of smart-devices intraconnected linking various streams together	Apple watch gathering step-count data as part of their exercise app
Effective data collection	Self-reported data gathering is useful in particular for healthcare professionals	Healthy; The self-care App which acts as diary cards for anyone with a health condition
Efficient data processing	Processing big data is useful to better understand the disease profile and potential treatment avenues	NHS Weight Loss Plan App acts as a support tool to anyone attempting to lose weight
Early diagnosis	Using data to make an early diagnosis through symptom tracking and/or identification of pattern inferences within gathered data is useful for healthcare systems, patients and clinicians	Sleepio App is used currently to support those with sleep-related issues. The app allows data to be gathered to personalise strategies to help improve sleep quality
Personalised treatment	Using data with patient preferences would aid development of personalised treatment	Various applications are currently available within a research context and are yet to be implemented clinically
